# Bacterial membrane vesicles transport their DNA cargo into host cells

**DOI:** 10.1038/s41598-017-07288-4

**Published:** 2017-08-01

**Authors:** Natalie J. Bitto, Ross Chapman, Sacha Pidot, Adam Costin, Camden Lo, Jasmine Choi, Tanya D’Cruze, Eric C. Reynolds, Stuart G. Dashper, Lynne Turnbull, Cynthia B. Whitchurch, Timothy P. Stinear, Katryn J. Stacey, Richard L. Ferrero

**Affiliations:** 1 Centre for Innate Immunity and Infectious Diseases, Hudson Institute of Medical Research and the Department of Molecular and Translational Sciences, Monash University, Clayton, Victoria 3168 Australia; 2 Department of Microbiology and Immunology, University of Melbourne, Parkville, Victoria 3010 Australia; 3 Monash Micro Imaging, Monash University, Clayton, Victoria 3168 Australia; 4 Ramaciotti Centre for EM, Monash University, Clayton, Victoria 3168 Australia; 5 Oral Health CRC, Melbourne Dental School, Bio21 Institute, The University of Melbourne, Parkville, Melbourne, Victoria 3052 Australia; 6 The ithree institute, University of Technology Sydney, Ultimo, NSW 2007 Australia; 7 School of Chemistry and Molecular Biosciences, University of Queensland, Brisbane Qld, 4072 Australia; 8 Biomedicine Discovery Institute, Department of Microbiology, Monash University, Clayton, Victoria 3168 Australia

## Abstract

Bacterial outer membrane vesicles (OMVs) are extracellular sacs containing biologically active products, such as proteins, cell wall components and toxins. OMVs are reported to contain DNA, however, little is known about the nature of this DNA, nor whether it can be transported into host cells. Our work demonstrates that chromosomal DNA is packaged into OMVs shed by bacteria during exponential phase. Most of this DNA was present on the external surfaces of OMVs, with smaller amounts located internally. The DNA within the internal compartments of *Pseudomonas aeruginosa* OMVs were consistently enriched in specific regions of the bacterial chromosome, encoding proteins involved in virulence, stress response, antibiotic resistance and metabolism. Furthermore, we demonstrated that OMVs carry DNA into eukaryotic cells, and this DNA was detectable by PCR in the nuclear fraction of cells. These findings suggest a role for OMV-associated DNA in bacterial-host cell interactions and have implications for OMV-based vaccines.

## Introduction

The release of small membrane vesicles (MVs) is a property that has been conserved by both multi- and unicellular organisms during evolution^1–6^. One of the major functions of these MVs is to facilitate intercellular communication and transport of molecules^7^. MVs typically contain many components of the parent cell. In Gram-negative bacteria, outer membrane vesicles (OMVs) were found to be enriched in many components of the outer membrane and periplasmic compartments^8^. Given the enrichment of specific membrane components and virulence factors within OMVs, it has been suggested that there may be selective packaging of cellular components within these structures^9–12^.

OMVs are produced during all stages of growth *in vitro* and *in vivo* ^13^, but are most abundantly produced in response to stress^14–16^. Although the mechanism of OMV biogenesis remains largely elusive, one hypothesis is that OMVs are formed through outer leaflet expansion, forming a membrane bulge which draws periplasmic contents into the vesicle before pinching off from the outer membrane^17, 18^. An interesting but as yet unexplained finding is the presence of cytoplasmic contents within OMVs, including DNA^19, 20^. A recent report of OMV production in *Pseudomonas aeruginosa* described a process of explosive cell lysis, in which spontaneously lysed bacteria release membrane fragments that form MVs and in so doing, engulf cytosolic contents, including DNA^21^. Since the first report in 1989, there have been a growing number of studies describing the presence of chromosomal and/or plasmid DNA in MVs^20, 22–24^. Nevertheless, the role(s) of OMV-associated DNA in host-pathogen interactions remain poorly defined.

It has been shown that OMVs can facilitate the inter- and intra-species exchange of DNA, thereby allowing the transfer of antibiotic resistance genes and virulence factors between bacteria^25–27^. OMV-associated DNA was also reported to play a role in the establishment of bacterial biofilms, thus aiding in host colonization^23, 28^. This suggests that OMV-associated DNA may be important in bacterial pathogenesis. Indeed, OMVs are known to efficiently enter eukaryotic cells and induce a range of cellular responses via lipopolysaccharide (LPS), proteins, toxins or peptidoglycan^29–33^. Although it naturally follows that the DNA cargo of OMVs is also likely to be transported into host cells, this has yet to be demonstrated formally.

In this study, we analyzed the DNA cargo of OMVs from five diverse Gram-negative pathogenic bacteria belonging to the Epsilonproteobacteria, Gammaproteobacteria and Bacteroidetes. We show that this OMV-associated DNA is mainly surface-located and is incorporated within the OMVs released by bacteria in the exponential phase of growth. Genomic analyses of *P*. *aeruginosa* OMVs revealed enrichment of specific chromosomal genes within the internal DNA. We demonstrate that bacterial DNA is carried into eukaryotic cells by OMVs and, moreover, could be detected by PCR within the nuclear fractions of these cells. In addition to providing a potential mechanism by which genetic material may be exchanged between prokaryotic and eukaryotic organisms, this study reveals a new perspective on the immunogenic properties of OMV-based vaccines.

## Results

### OMVs from a range of Gram-negative pathogens carry DNA externally and internally

**Figure 1:**
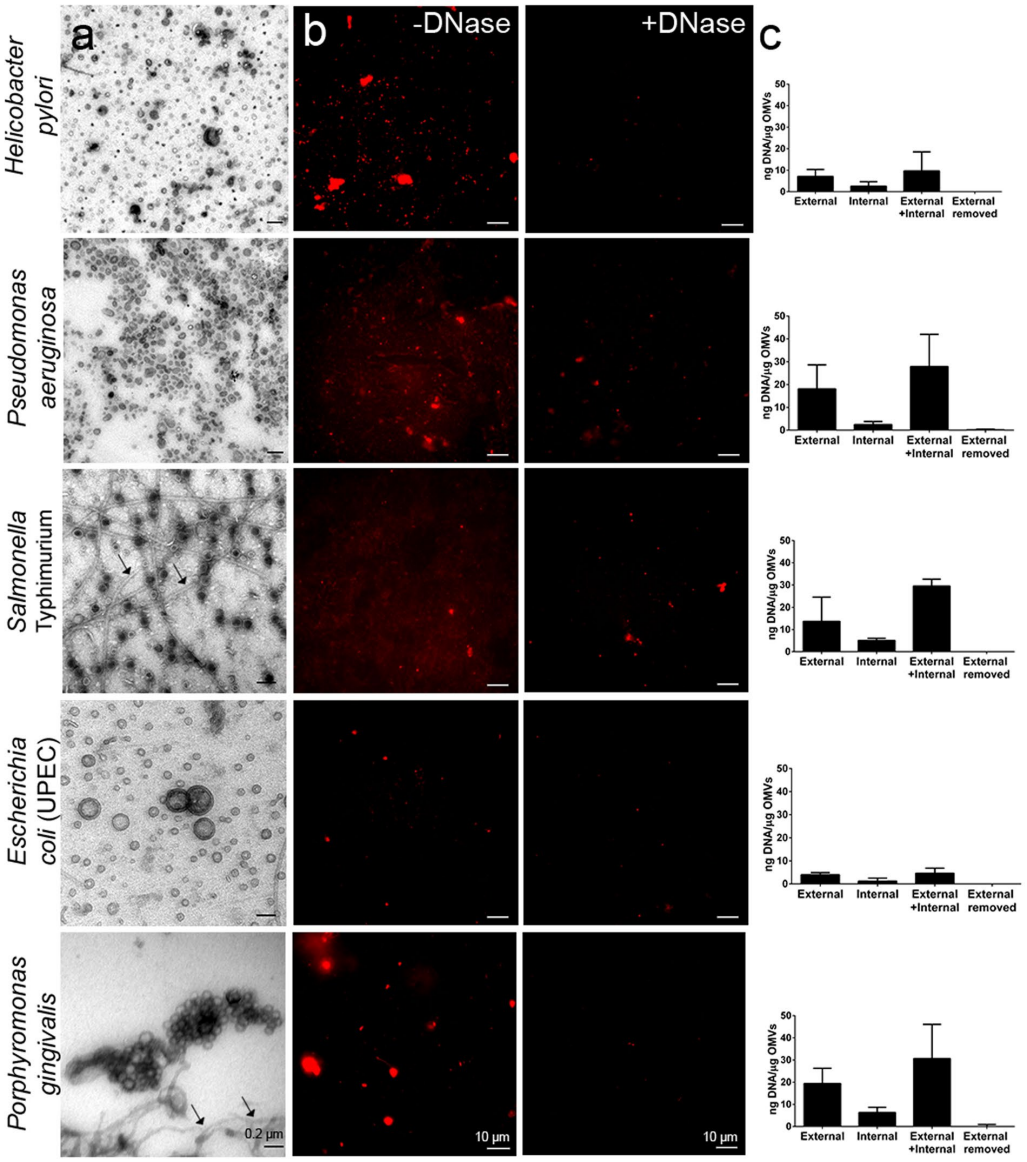
OMVs from Gram-negative bacteria carry DNA. (**a**) Transmission electron microscopy (TEM) of OMVs from Gram-negative bacteria (70× magnification, scale bar = 0.2 μm). Arrows indicate the presence of flagella in the *S*. Typhimurium and *P*. *gingivalis* OMV preparations. (**b**) Confocal images of OMVs treated with and without DNase and stained with the membrane permeable DNA stain SYTO-61, scale bar = 10 μm. (**c**) Quantification of internal and external OMV-associated DNA by Quant-iT PicoGreen dsDNA assay (n = 3). Complete removal of external DNA was verified by quantifying the DNA on intact DNase-treated OMVs (external removed).

The presence of DNA has been reported in OMV preparations from an increasing number of Gram-negative bacteria^20, 22–24^. To investigate whether this may be a broad feature of Gram-negative bacteria, we isolated OMVs from exponential phase cultures of five diverse Gram-negative pathogens (Fig. 1a). DNA was detected by SYTO-61 nucleic acid staining associated with the OMVs of all species examined (Fig. 1b). Agarose gel electrophoresis and ethidium bromide staining revealed high molecular weight bands consistent with genomic DNA (gDNA) in all OMV preparations, except those from *Helicobacter pylori* and uropathogenic *Escherichia coli* (UPEC), in which the amounts of DNA were below the limit of detection (Supplementary Fig. 1). Most of the OMV-associated DNA could be removed by DNase treatment, suggesting a predominantly external location for this material (Fig. 1). Interestingly, a smaller molecular weight band of approximately 3 kb was present in the *Salmonella enterica* Typhimurium OMVs and remained after DNase-treatment, suggesting that these OMVs may carry plasmid both externally and internally (Supplementary Fig. 1).

To confirm these findings, external and internal DNA concentrations in OMVs were quantified using the Quant-iT PicoGreen dsDNA assay (Fig. 1c). As PicoGreen reagent is not membrane permeable, it was possible to achieve complete removal of the external DNA after treating intact OMVs with DNase (Fig. 1c; “external removed”). Thus, to quantify internal DNA, OMVs were first treated with DNase, then lysed with guanidinium thiocyanate, prior to analysis using the Quant-iT PicoGreen assay. Total DNA concentrations were measured by lysing OMV samples that had not previously been treated with DNase (see Methods). We showed that the DNA concentrations on the external surfaces of OMVs were greater than those within OMVs for all bacterial species (Fig. 1c). The relative amounts in each compartment, however, varied between bacterial species. The largest amount of external DNA was found on OMVs from *S*. Typhimurium (23.7 ng DNA per μg OMV ± 0.4 SEM), *Porphyromonas gingivalis* (19.4 ng DNA per μg OMV ± 1.6 SEM) and *P*. *aeruginosa* (18.1 ng DNA per μg OMV ± 0.3 SEM). Internal DNA levels were similar across the different bacterial species, with an average of 3.54 ng DNA per μg OMVs ± 0.99 SEM. Sucrose gradient fractionation of *P*. *aeruginosa* OMV preparations showed that the DNA was mainly present in fractions 4–6, corresponding to smaller-sized OMVs of approximately 20 nm (Supplementary Fig. 2), which is consistent with previous data^33^.

**Figure 2:**
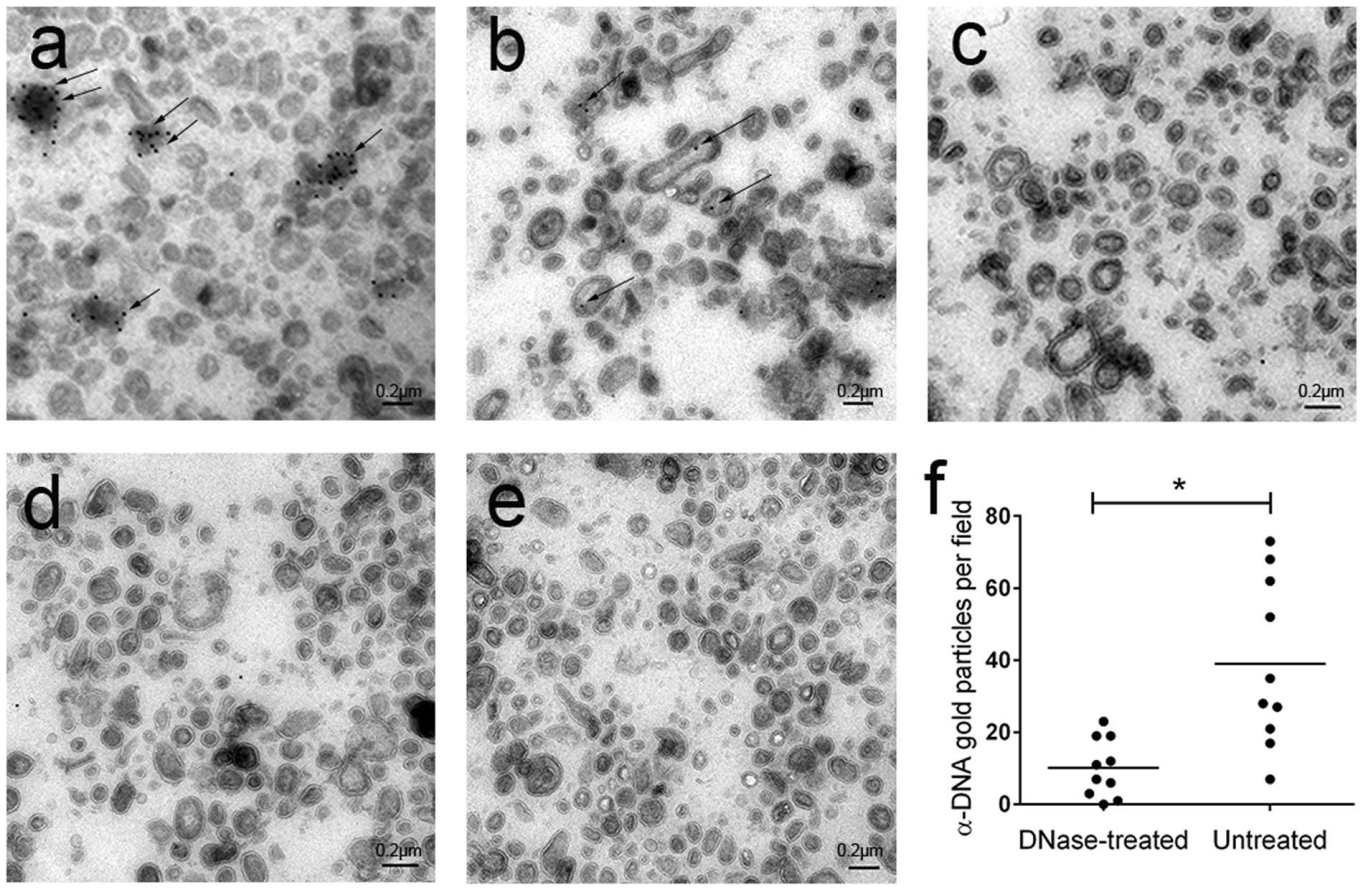
*P*. *aeruginosa* OMVs are associated with DNA external and internal to the vesicle membrane. TEM of (**a**) Untreated OMVs labeled with anti-DNA antibody. Both internal and external OMV-associated DNA is observed (indicated by arrows). (**b**) DNase-treated OMVs labeled with anti-DNA antibody showing internal OMV-associated DNA only. (**c**) DNase treatment post-sectioning to remove exposed internal OMV-associated DNA, followed by detection with anti-DNase antibody. (**d**) Secondary antibody and protein-A-gold (PAG) only and (**e**) PAG only. Scale bar = 0.2 μm; 100× magnification. (**f**) Quantification of α-DNA labeling of DNase-treated versus untreated OMVs (p = 0.0012, n = 10 fields of view).

Transmission electron microscopy (TEM) was used to confirm the presence of external and internal OMV-associated DNA. Untreated and DNase-treated *P*. *aeruginosa* OMVs were embedded in gelatin and sectioned into thin sections to expose both the external and internal OMV-associated DNA (see Methods). Immunogold labeling with an anti-DNA antibody detected DNA on the outside and inside of untreated OMVs (Fig. 2a). DNase-treatment significantly reduced the levels of gold labeling on OMVs, thus confirming that most of the DNA in bacterial OMVs was present on the surface of these structures (Fig. 2b,f). Nevertheless, some DNA clearly was also present internally. DNase treatment of sectioned samples showed complete removal of both external and internal OMV-associated DNA (Fig. 2c). Specificity of the antibody was confirmed using secondary antibody and protein-A-gold controls (Fig. 2d,e). The finding that OMVs contain DNA within the vesicle lumen is consistent with the recent report from Perez-Cruz *et al*.^20^.

### Bacterial DNA becomes associated with OMVs released during exponential growth

**Figure 3:**
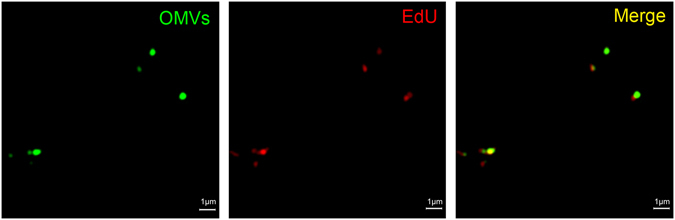
Super resolution microscopy of OMVs carrying bacterial DNA. OMVs were labeled with the lipophilic stain, DiO (green), which targets the OMV membrane, while EdU-incorporated DNA (red) was labeled by a 2-step process. The first step involved incubation with 12.5 μM biotin azide, during which the azide moiety was bound specifically to the alkyne backbone of the EdU molecule in the presence of a copper catalyst. The second step involved incubation with 5 μg/ml streptavidin-conjugated 568 Alexa Fluor^®^ to fluorescently label the biotin-azide bound to the EdU. Scale bar = 1 μm.

In order to develop a specific labeling technique for OMV-associated DNA, we adapted the Click-iT EdU assay (Molecular Probes) to label nascent DNA within dividing bacteria (Supplementary Figs 3 and 4). EdU-labeling of DNA in OMVs was optimal for bacteria grown to mid-exponential phase (4 h incubation; Supplementary Fig. 4a,b). Using super resolution microscopy, we demonstrated co-localization of this EdU-labeled DNA with DiO-labeled OMV membranes (Fig. 3). These results confirm that bacterial DNA is associated with OMVs released by actively dividing bacteria.

### Molecular characterization of OMV-associated DNA

To determine for the first time the molecular characteristics of DNA located within OMVs, we isolated the total and internal-only DNA fractions from five biological replicates of *P*. *aeruginosa* PA103 OMVs. As a control for the possible selective enrichment of specific genetic regions in sequencing “reads”, gDNA from *P*. *aeruginosa* PA103 was also sequenced. To exclude potential DNA contamination from viable or non-viable bacteria, OMV preparations were analyzed by TEM, confocal microscopy and viable counts prior to DNA isolation (Supplementary Fig. 5a–c). Total OMV-derived DNA was isolated from intact OMVs and is therefore predominantly representative of the external OMV-associated DNA, as our previous findings in this study demonstrate (Fig. 1c). Internal OMV-derived DNA was isolated from DNase-treated OMVs from which all external DNA had been removed (Fig. S5d). Purified internal DNA could be visualized by agarose gel electrophoresis (Supplementary Fig. 5e).

**Figure 4:**
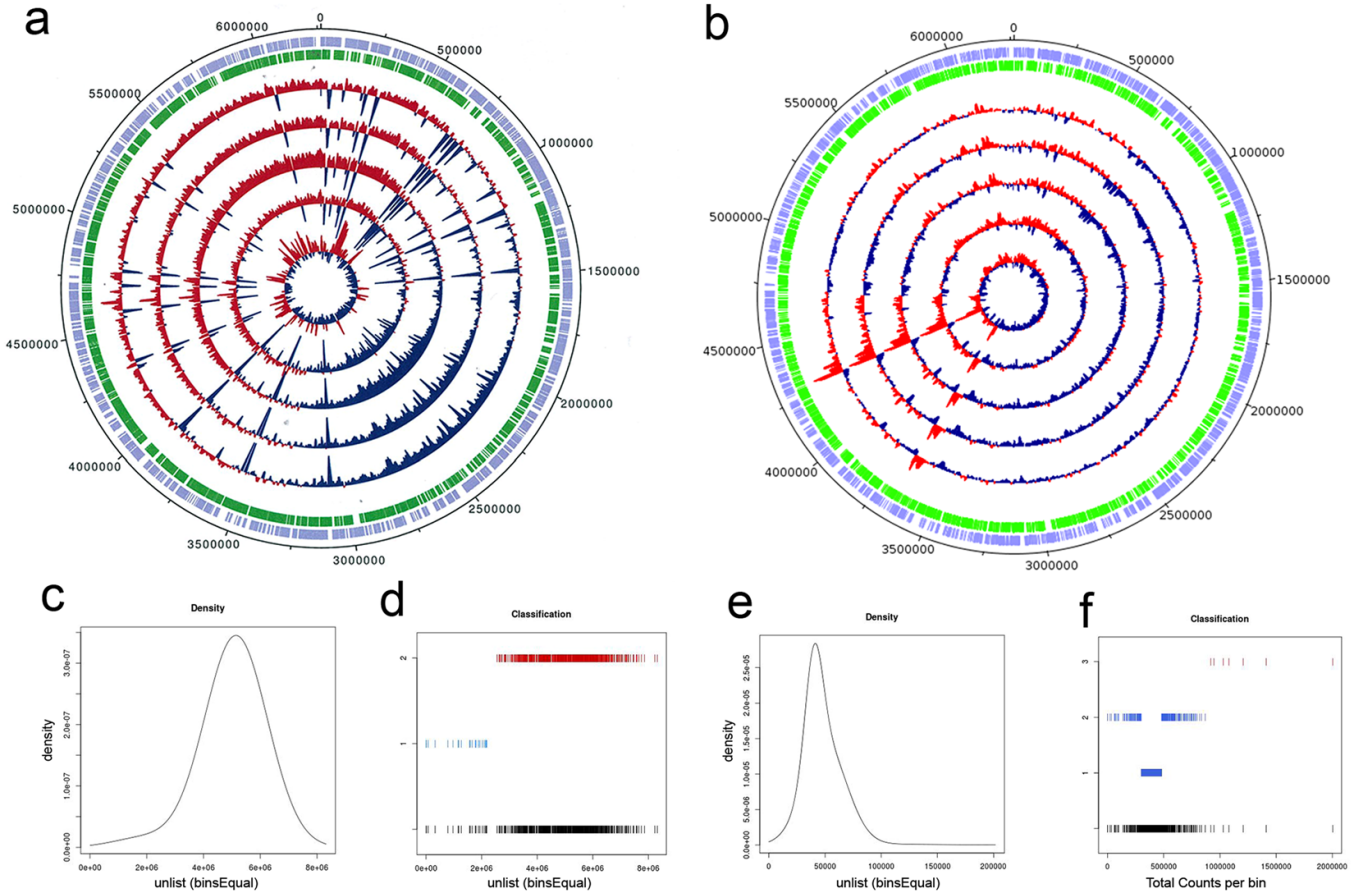
Analysis of total and internal OMV-derived DNA sequences from five separate biological replicates of *P*. *aeruginosa* OMVs. Circular plots for the total (**a**) and internal (**b**) DNA read count across each of the replicates show read count at each base pair position. Positions showing greater than average read density are coloured red while those with less than average density are coloured blue. A peak in read count density is visible in the internal DNA at approximately 4,290,000 bp. The outer circles show the location of the genes on the positive (purple) and negative (green) strands of the reference genome. The total read counts from 603 equally-sized and contiguous bins spanning the genome were computed and plotted as density distributions for the total (**c**) and internal (**d**) DNA. A Kolmogorov-Smirnov test revealed that the read count distribution for the total DNA did not differ from a normal distribution, whereas the internal DNA possessed a long tail at the upper end of the distribution which created a significant deviation from the normal distribution (p = 1.382 × 10^−6^). The Gaussian finite mixture models confirmed these observations (**d**,**f**). The counts from the majority of the total DNA bins were contained within a dominant Gaussian distribution (red), while a secondary Gaussian distribution represented a small subset of bins with relatively low read densities (blue) (**d**). In contrast, the counts for the internal DNA bins were represented by three Gaussian distributions: two overlapping distributions (blue) represented the majority of bins with counts ranging from 0 to approximately 800 000; and a third Gaussian distribution (red) represented bins with counts at higher values (**f**).

Next-generation sequencing revealed that sequences from the total, internal and gDNA preparations mapped fully to the reference genome (Fig. 4a,b and Supplementary Fig. 6). As a fully closed genome sequence for *P*. *aeruginosa* PA103 is not available, all sequencing data were mapped to that of *P*. *aeruginosa* PAO1 (ATCC 47085). To search for enrichment, each sequence was divided into equally-sized contiguous bins and the number of reads per bin was determined. The read count for each bin was then plotted and the distribution of the curve fitted to the Gaussian finite mixture model. This revealed that the read counts observed in the total DNA sequence followed a normal distribution, with no obvious regions of enrichment (p = 0.143; Fig. 4c,d). In contrast, the internal DNA reads did not follow a normal distribution, with a distinct group of over-represented, or enriched bins which was highly significant (p = 1.382 × 10^−6^; Fig. 4e,f). This enriched region was located close to 4,300,000 bp (Fig. 4a) and encompassed 35 known or putative genes (Table S1). Bioinformatic analysis of the gDNA did not show the same pattern of enrichment (Supplementary Fig. 6). The known genes encoded several virulence-related products, including ExoS cytotoxin (*exoS* ^34^) and its chaperone SpcS (*spcS* ^35^), a biosurfactant (*rlhB* ^36^) and the membrane nitrite reductase operon (*narGHIJ*, *K1*, *K2* ^37^). The gene products encoded by this operon are linked to *P*. *aeruginosa* pathogenesis through their regulation of type III secretion system functions, motility and biofilm formation^38^. Furthermore, genes were identified with roles in bacterial survival under stress conditions (*capB* ^39^), antibiotic resistance (*prc* ^40^), metabolism (*dauA*, *daub* ^41^) and membrane synthesis (*pcs* ^42^). These data were highly reproducible across the five biological replicates. Our results show that the DNA within these OMVs is chromosomal in nature and that specific genes are enriched in the internal compartments of *P*. *aeruginosa* OMVs.

### OMV-derived DNA is detected in the nuclear fraction of epithelial cells

**Figure 5:**
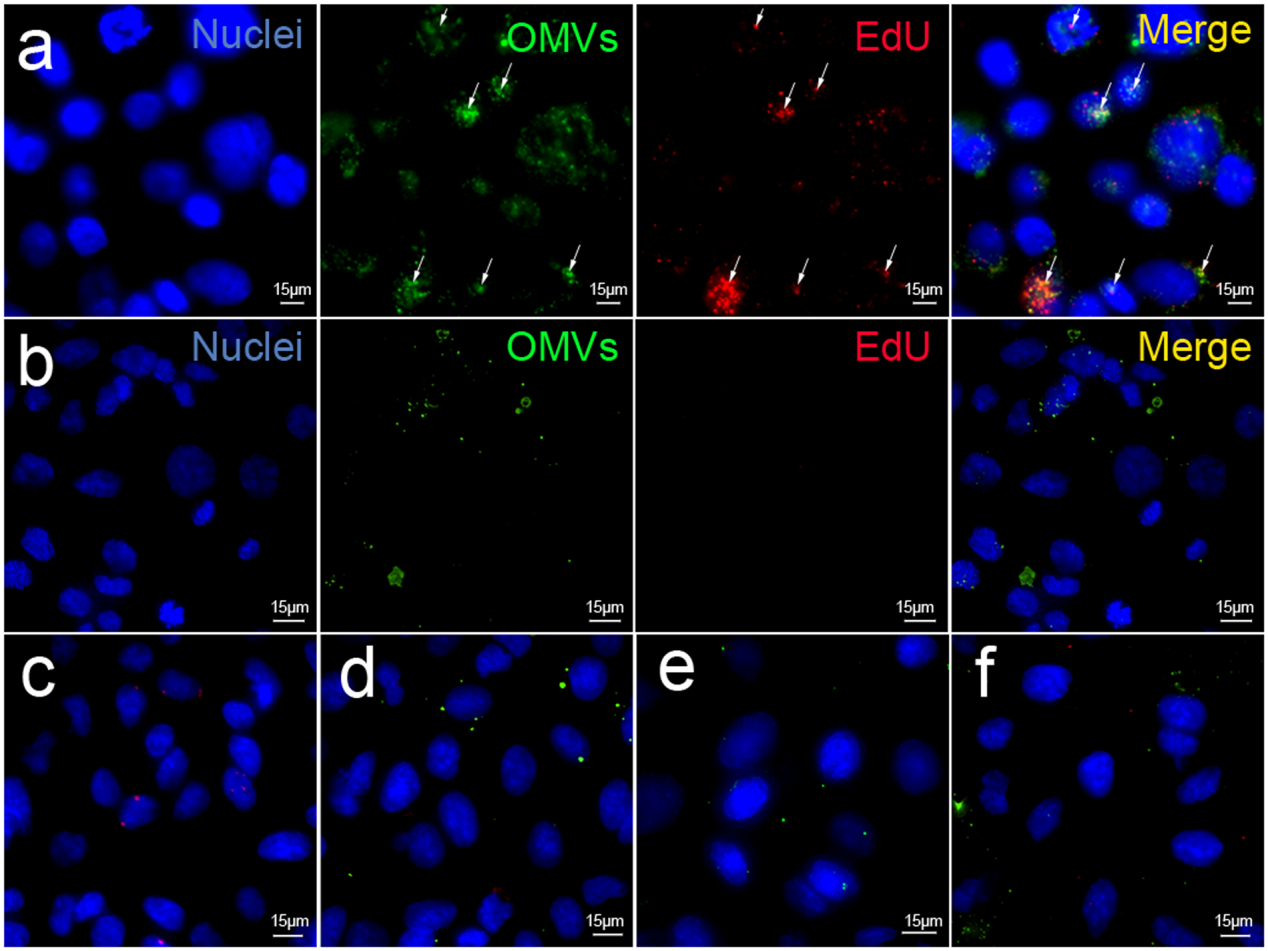
Confocal microscopy showing internalization of OMV-associated DNA in A549 lung epithelial cells. EdU-labeled OMVs, either (**a**) untreated or (**b**) DNase-treated, were labeled with the lipophilic stain DiO then added to A549 cells for 5 h, washed to remove non-internalized OMVs, fixed and permeabilized. EdU was detected in a 2-step reaction consisting of incubation with biotin azide, in which the azide binds specifically to the alkyne backbone of EdU, followed by fluorescent detection with streptavidin-conjugated Alexa-Fluor 568. Co-localization of DiO (green) and EdU (red) labels in internalized OMVs are indicated by arrows. Nuclei are stained with DAPI (blue). Merged images showing control samples in which either: (**c**) biotin azide and streptavidin-AlexaFluor 568 only, (**d**) no biotin azide or (**e**) DiO only were added to cells. (**f**) Non-permeabilized cells. Scale bar = 15 μm.

It has been shown that OMVs play a role in long distance delivery of DNA to other bacteria, including other bacterial species^31^. To determine whether OMVs have the ability to deliver DNA into eukaryotic host cells, we added EdU-labeled *P*. *aeruginosa* OMVs to A549 lung epithelial cells. EdU-labeled DNA was shown to co-localize with DiO-labeled OMV membrane within the epithelial cells (Fig. 5). This was also observed in a gastric epithelial cell line (Supplementary Fig. 7).

**Figure 6:**
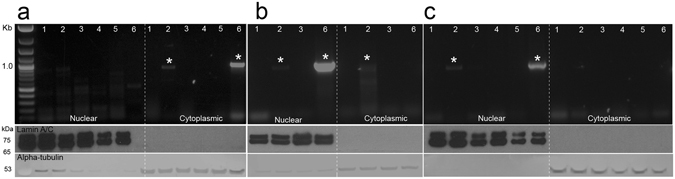
DNA derived from *P*. *aeruginosa* OMVs is detected in the nuclear fractions of epithelial cells. A549 cells were either left untreated (1), treated with intact OMVs (2), DNase-treated OMVs (3), disrupted OMVs (4), free *P*. *aeruginosa* gDNA (5), or plasmid pGL3c (6) transfected with Lipofectamine 2000 (Life Technologies). After treatment for (**a**) 4 h, (**b**) 8 h and (**c**) 18 h, cells were DNase-treated to remove non-internalized DNA, followed by extraction of cytoplasmic and nuclear compartments. PCR products, corresponding to OMV-derived DNA and pGL3c (859 bp and 966 bp, respectively) are indicated by asterisks. Nuclear and cytoplasmic fractions were confirmed by Western blotting using anti-lamin A/C and -alpha tubulin antibodies, respectively.

We next investigated the intracellular trafficking of OMV-derived DNA in epithelial cells. For this, OMVs and control samples were added externally to cells and DNA trafficking over time monitored by PCR. Non-internalized, extracellular DNA was removed by DNase treatment of cells. The isolation of nuclear and cytoplasmic fractions was confirmed by Western blotting using anti-lamin A/C and -alpha tubulin antibodies, respectively (Fig. 6a–c). Consistent with the findings for transfected pGL3c plasmid, we detected OMV-derived DNA in the cytoplasmic but not the nuclear fractions of cells at 4 h (Fig. 6a). Importantly, OMV-derived DNA was detected in nuclear fractions at 8 h and 18 h (Fig. 6b,c). In contrast, trafficking of OMV-derived DNA was not observed in cells incubated with either DNase-treated or disrupted OMVs (Fig. 6). Free gDNA was not taken up by the cells, confirming that OMVs are required to transport DNA into the cells (Fig. 6). Endosomal contamination was excluded as a potential source of OMV-derived DNA in nuclear fractions (Supplementary Fig. 8). Taken together, these data indicate that *P*. *aeruginosa* OMVs and their DNA cargo enter eukaryotic cells. Moreover, the preliminary evidence suggests that OMV-associated DNA may localize to the nucleus of these cells.

## Discussion

Eukaryotic MVs have been extensively studied for their nucleic acid content (DNA, RNA, microRNA) and biological importance in various diseases^43^. In contrast, much less is known regarding the properties and functions of DNA in prokaryotic MVs. The present study establishes that DNA is present on the internal and external surfaces of OMVs from a diverse range of Gram-negative pathogens and describes a detailed molecular characterization of the DNA associated with OMVs. A key finding of the present work was the observation that DNA is carried into non-phagocytic host cells via OMVs and that this DNA was detectable by PCR in the nuclear fraction of cells.

Previous studies demonstrated the presence of DNA in OMVs purified from bacterial culture supernatants^21, 24, 44^, however, it remained ambiguous whether the DNA was actively “secreted” as part of the OMVs or simply released during cell lysis. To explore this, we utilized the Click-iT EdU assay to specifically label nascent DNA in bacterial cultures. We showed that bacterial gDNA becomes associated with OMVs during exponential phase growth (Fig. 3, Supplementary Fig. 4a,b). It has recently been shown that OMVs can form as a result of spontaneous cell lysis, and that these OMVs harbor DNA^21^. Although we cannot exclude that some OMV-associated DNA is the result of spontaneous cell lysis, it appears that DNA is actively shed within the OMVs released by dividing bacteria. Consistent with this suggestion, Liao *et al*.^23^ reported that MVs from the Gram-positive bacterium *Streptococcus mutans* carry 2.82-fold more DNA when harvested from early-exponential phase than overnight cultures. This suggests a difference in the mode of MV production in exponential phase growth when compared with stationary phase growth.

Consistent with recent work^20, 22, 45^, we showed that most of the DNA in OMVs is present externally, however, our study demonstrates that OMVs also carry DNA internally (Figs 1 and 2). Next generation sequencing of the total OMV-derived DNA, which comprised mainly external DNA, showed that the OMVs were surrounded by gDNA (Fig. 4a). This is consistent with a recent study in which the total OMV-derived DNA from *P*. *aeruginosa* PAO1 was sequenced and shown to cover the full genome^21^. Our genomic analyses of the internal DNA fractions of OMVs from five independent biological replicates revealed two small genomic regions of 1.09 Kb and 4.16 Kb that were consistently present in higher abundance in these fractions when compared with total OMV-associated DNA and gDNA preparations (Fig. 4a). Biller *et al*.^22^ also reported a high relative abundance of selected regions in the internal DNA isolated from OMVs of the marine cyanobacterium *Prochlorococcus*, but did not identify the genes. In the current study, we show that the internal OMV-derived DNA from the pathogen *P*. *aeruginosa* is selectively enriched in genes involved in a variety of biological functions (Table S1). This DNA may be selectively packaged to enable horizontal gene transfer, thereby conferring a competitive advantage to the bacterium. OMVs from a variety of bacterial species have been shown to be involved in the horizontal transfer of antibiotic resistance or virulence genes^24–27, 46^. Although it is not clear whether this genomic DNA can be integrated and expressed in a recipient bacterium, our data suggests that the DNA internal to OMVs may promote gene sharing between bacteria. In contrast, the DNA on the external surfaces of OMVs appears to be important for biofilm formation and protection of the biofilm^23, 25, 28^. Thus, we propose that external and internal OMV-associated DNA play differing roles in bacterial cell-cell communication.

OMVs are able to enter eukaryotic cells and deliver their protein, LPS and peptidoglycan cargos into host cells^8^. By fluorescently labeling the DNA within OMVs, we showed that OMVs can also carry their DNA cargo into eukaryotic cells (Fig. 5, Supplementary Fig. 8). Furthermore, PCR analysis of cytoplasmic and nuclear fractions of A549 cells treated with OMVs suggested possible trafficking of OMV-derived DNA through the cytosol and to the nucleus or perinuclear space of eukaryotic host cells (Fig. 6). Consistent with our findings, Rompikuntal *et al*.^47^ reported that *Aggregatibacter actinomycetemcomitans* OMVs localize to the nuclear or perinuclear space and deliver cytolethal distending toxin (CDT) into the nucleus of host cells. Similar results have been observed with eukaryotic extracellular vesicles, which have been shown to contain gDNA fragments that are transported into the host cell nucleus, resulting in increased mRNA specific to the transferred genes^48^. Although the presence of OMV-derived DNA in the nucleus needs to be definitively proven, it is possible that OMVs could have a similar effect on host cells. Indeed, there is evidence suggesting that transfer of bacterial genetic material occurs in human somatic cells, with integrations of bacterial DNA detected in the host genome^49^. These bacterial sequences were detected more frequently in stomach tumor cells compared with non-tumor cells, and the most prevalent bacterial DNA detected was from *Pseudomonas* spp.^49^. However, the mechanism behind this foreign DNA integration is uncharacterized and it remains to be determined whether OMV-derived DNA may be a source of integrated bacterial DNA. Future studies will be directed at confirming the ability of OMVs to deliver DNA to the nucleus and the potential of this material to integrate into the host genome or modulate the innate immune response via one or more DNA sensors^50^.

In summary, our data demonstrate that OMV-associated DNA is not only important for inter-bacterial communication, but may also play a role in host-pathogen interactions. We suggest that OMV-associated DNA may modulate host cell responses and thus the findings have implications for the use of OMVs as vaccines.

## Methods

### Bacterial strains and growth conditions

*H*. *pylori* 251^33^, *P*. *aeruginosa* PA103 Δ*pilA* ^51^, *S*. Typhimurium SL1344^52^, UPEC CFT073^53^ and *P*. *gingivalis* W50^54^ were all grown as described previously.

### Cell culture strains and growth conditions

AGS gastric adenocarcinoma cells (ATCC CRL1739) and A549 adenocarcinoma human alveolar basal epithelial cells (ATCC CCL185) were maintained in RPMI medium (Gibco) supplemented with 10% fetal calf serum, 1% L-glutamine and 1% penicillin/streptomycin^29, 33^. Phenol red-free RPMI 1640 (Life Technologies) was used for imaging.

### Isolation and purification of OMVs

Mid-exponential phase cultures were pelleted at 4,000 × g for 40 min at 4 °C (Heraeus Multifuge 3SR, ThermoScientific). Supernatants were filtered through 0.22-µm-pore-size filters (Millipore). OMVs were isolated by ultracentrifugation, as described previously^33, 55^. The pellets were washed in PBS by ultracentrifugation, re-suspended in PBS and stored at −80 °C. OMV protein concentrations were quantified using the Bradford Protein Assay (BioRad).

### Sucrose gradient fractionation of OMVs

OMVs were washed three times in PBS in Amicon YM-10 columns (Millipore) and layered on to a discontinuous sucrose gradient (25, 42 and 56% w/v). Separation was achieved by ultracentrifugation at 100,000 × g for 16 h as described previously^33, 55^. Protein and DNA concentrations of each 3 ml fraction was measured using Qubit^TM^ fluorometric quantification (Invitrogen). Protein profiles of fractions were assessed by Western Blot, while DNA was visualized on a 0.8% agarose gel stained with SYBR^®^ Safe (Life Technologies).

### Fluorometric DNA quantification

External and internal OMV-associated DNA was quantified using the PicoGreen assay (Molecular Probes) as described^24^. OMVs were treated with or without 2 U of TURBO^®^ DNase I (Ambion) to remove external OMV-associated DNA. Samples were then treated with or without GES lysis reagent (5 M guanidinium thiocyanate, 100 mM EDTA, 0.5% v/v Sarkosyl) to release internal DNA. Samples were assayed according to the manufacturer’s instructions and fluorescence measured in a FLUOstar OPTIMA plate reader (BMG Labtech).

### OMV-associated DNA extraction and sequencing

All traces of bacteria were removed from *P*. *aeruginosa* cultures by pelleting at 4,000 × g for 40 min, at 4 °C. Supernatants were transferred to a fresh tube, centrifuged three times and filtered three times through 0.22-µm-pore-size filters. OMVs were pelleted from the cell-free supernatant by ultracentrifugation. OMV pellets were re-suspended in PBS and washed by ultracentrifugation to remove extracellular DNA not associated with the OMVs. One tenth of the final volume of the recovered OMVs was plated onto antibiotic-free LB agar to confirm the absence of viable bacteria. The absence of viable and non-viable bacteria was further verified by thorough examination by electron microscopy and confocal microscopy. Total OMV-associated DNA was isolated from intact OMVs. Internal OMV-associated DNA was isolated by removing external DNA using 2 U of TURBO^®^ DNase I (Ambion) according to the manufacturer’s directions, followed by release of the internal DNA by lysis of the vesicles with GES reagent. DNase was inactivated at 75 °C for 15 min. Quant-iT PicoGreen dsDNA assay (Invitrogen) was used to verify complete digestion of external OMV-associated DNA. DNA was purified by phenol-chloroform purification and precipitated in 2 volumes 100% ethanol and one tenth volume 0.3 M Na acetate (pH 5.0). RNA was removed with RNase ONE^TM^ (Promega) and salts removed using PureLink^®^ gDNA purification kit (Life Technologies).

Initial sequencing was carried out on Ion Torrent PGM (Life Technologies) at the MHTP Medical Genomics Facility. Sequence libraries were constructed with the Ion Xpress™ Plus gDNA Fragment Library kit with DNA (90 ng). Enriched template libraries were sequenced on the Ion Torrent PGM (Life Technologies) using the Ion PGM Sequencing 400 bp Kit and Ion 318™ Chip Kit v2. Subsequent sequencing replicates were performed on the NextSeq platform (Illumina). DNA libraries were constructed using the Nextera XT DNA preparation kit (Illumina) and sequencing was carried out using 2 × 150 bp paired-end chemistry. The Samtools pileup tool was used to convert the Bam files into read counts at each position for internal and external OMVs. The data were then loaded into the DNAplotter software^56^ to create circular plots showing read count number (hits) at each base position along with gene location and GC content. The null hypothesis that the distribution of hits across 630 contiguous and equal sized genomic bins in the internal OMV DNA conformed to a single normal distribution was tested using the Kolmogorov-Smirnov test using the R statistical software (https://www.R-project.org). Gaussian finite mixture models were fitted to identify multiple Gaussian distributions within the population of hits from the bins using the Mclust R package. The genomic regions from the distribution with the greatest hits were selected as representing regions of gDNA that were enriched within the OMVs. A custom Perl script was used to extract the genes present from within the enriched regions of gDNA.

### Click-iT EdU labeling of OMV-associated DNA

OMV-associated DNA was labeled using the Click-iT EdU kit (Molecular Probes). Briefly, *P*. *aeruginosa* bacteria were cultured in tryptic soy broth (TSB; Bacto, BD) containing 1.2 mM of EdU (5-ethynyl-2-deoxuridine), a nucleoside analogue of thymidine, for 4 h on an orbital shaker (225 rpm) at 37 °C. The EdU-labeled OMVs were isolated as described above and added to epithelial cells in 8-well chambers (Ibidi). After the required incubation, cells were washed twice in PBS before fixing in 4% paraformaldehyde (PFA) and permeabilization in 3% Triton-X 100. Click-iT EdU labeling involves a two-step process. The first step involves incubation with 12.5 μM biotin azide (Molecular Probes), during which the azide moiety binds specifically to the alkyne backbone of the EdU molecule in the presence of a copper catalyst. The second step involves incubation with 5 μg/ml streptavidin-conjugated 568 Alexa Fluor^®^ (Molecular Probes) to enable fluorescent detection of the biotin-azide bound to the EdU.

### Fluorescence microscopy

OMVs were labeled with the lipophilic fluorescent dyes DiO or DiI (Molecular Probes; 1:100) for 30 min at 37 °C. Labeling of OMV-associated DNA was carried out using either SYTO^®^-61 (Molecular Probes; 1:1000), Click-iT EdU (as above) or mouse anti-DNA IgM monoclonal antibody (Novus Biologicals; 1:20). OMVs were washed four times with PBS using Amicon YM-10 columns. Labeled OMVs (50 µg/mL^33^) were added to epithelial cells. Cells were washed twice in PBS before fixing in 4% PFA. Samples were mounted in VectaShield mounting medium (Vectorlabs). Microscopy was performed using a Deltavision deconvolution microscope (60 ×, 1.42 objective). Image analysis was carried out using Imaris ×64 7.6.5.

### Super resolution microscopy (SRM)

OMVs isolated from cultures grown in the presence of EdU (described above) were coated onto coverslips pre-treated with poly-L-lysine (Sigma), EdU Click-labeled and stained with the lipophilic dye DiO (Molecular Probes). Coverslips were mounted onto slides with VectaShield (Vectorlabs). SRM was performed on a DeltaVision OMX 3D-SIM. Image analysis was carried out using Imaris ×64 7.6.5.

### Transmission electron microscopy

Formvar- and carbon-coated TEM grids were pre-incubated with poly-L-lysine. TEM grids were placed on top of a 10 μL droplet of OMVs at a concentration of 1 µg/µL (10 min) and washed with PBS (2 × 10 min). Samples were subsequently fixed in 1% glutaraldehyde·PBS (5 min), followed by dH_2_O washes (7 × 1 min). Vesicles were stained with 2% uranyl oxalate pH 7.0 (5 min) and methylcellulose uranyl acetate (10 min). Samples were viewed using a Hitachi H-7500 transmission electron microscope equipped with Gatan Multiscan 791 camera.

### Preparation of OMV ultrathin sections and immunogold labeling

OMV samples were treated with or without TURBO^®^ DNase I (Ambion) then fixed in 4% PFA and prepared accordingly^57^. Ultrathin cryo-sections were transferred to carbon-coated formvar grids and incubated with mouse monoclonal IgM anti-dsDNA antibody (1:2; Novus Biologicals)^58^. Samples were viewed using a Hitachi H-7500 transmission electron microscope equipped with Gatan Multiscan 791 camera.

### Detecting OMV-associated DNA internalized by epithelial cells

*P*. *aeruginosa* OMVs (65 μg protein, containing 1 μg of OMV-associated DNA) were treated or not with TURBO^®^ DNase I (Ambion). As controls, cells were treated with either: OMVs disrupted by sonication 3 times 30 s each and 3 freeze-thaw cycles; *P*. *aeruginosa* gDNA (1 μg); or pGL3c-control vector (100 ng; Promega) transfected with Lipofectamine 2000 (Life Technologies). Treatments were added to A549 cells (5 × 10^5^/mL) in 6-well plates. After incubation for 4–18 h, cells were washed three times with PBS and treated with DNase to remove non-internalized DNA, then thoroughly washed to remove DNase. Cytoplasmic and nuclear fractions were separated using a Nuclear Extraction Kit (Active Motif) according to the manufacturer’s instructions. DNA was precipitated from extracts in ethanol/sodium acetate and used in PCR reactions with primers targeted the *P*. *aeruginosa narG* gene (5′-TGTTCAACAGTTTCTCGGTGGTC-3′ and 5′-GAGAAGGACGACCTCAACACCTC-3′; product 859 bp) or pGL3c plasmid (5′-CGTTCGGTTGGCAGAAGCTA-3′ and 5′-AGCGTTTTCCCGGTATCCAG-3′); product 966 bp. Products were visualized on 1% agarose gels stained with SYBR^®^ Safe (Life Technologies). Proteins were resolved on NuPAGE^®^ 4–12% Bis-Tris Protein Gels (Invitrogen) using NuPAGE^®^ MES SDS running buffer (Invitrogen). Proteins were transferred onto nitrile membranes using the iBlot^®^ Gel Transfer System (Life Technologies) for 7 min. Membranes were blocked in 5% (w/v) milk powder in TBST-T (50 mM Tris-HCL, pH 7.6, 150 mM NaCl and 0.1% (v/v) Tween-20) or Odyssey Buffer (LI-COR), as appropriate. The nuclear marker mouse anti-lamin A/C (Cell Signalling; 1:1,000) was used, followed by rabbit anti-mouse-HRP secondary antibody (Dako, 1:2,000). The cytoplasmic marker rat anti-alpha tubulin (Abcam; 1:1,000) was used, followed by goat anti-rat-IRDye800 secondary antibody (Rockland, 1:3,000). Endosomes were detected by Western blotting using goat anti-EEA1 (Santa Cruz; 1:1,000), followed by rabbit anti-goat-HRP (Dako; 1:2,000) and mouse anti-LAMP1 (BD Bioscience; 1:1,000), followed by rabbit anti-mouse Alexa Fluor^®^ 680 (Molecular Probes; 1:1,000). Membranes were developed using LumiGLO reagent (Cell Signaling) or by the Odyssey Infrared Imaging System (LI-COR Bioscences).

For TEM analysis, A549 cells were treated with OMVs for 4 h or 18 h, fixed in 4% PFA and prepared accordingly^57^. Ultrathin cryosections were transferred to carbon-coated formvar grids and viewed on a Hitachi H-7500 transmission electron microscope equipped with Gatan Multiscan 791 camera. Fluorescence microscopy images were obtained as described above using DiI-labeled OMVs added to A549 cells for 8 h.

## Electronic supplementary material

Supplementary Information
