# Supplementary material for: Bacterial membrane vesicles transport their DNA cargo into host cells
Source: Sci Rep. 2017 Aug 1;7:7072. doi: 10.1038/s41598-017-07288-4 (PMC5539193; doi:10.1038/s41598-017-07288-4)
Supplement: Supplementary file 1 — Supplementary Information [file 41598_2017_7288_MOESM1_ESM.pdf]

Supplementary Information

Bacterial membrane vesicles transport their DNA cargo into host cells

Natalie J. Bitto[1,2], Ross Chapman[1], Sacha Pidot[3], Adam Costin[4,5], Camden Lo[4], Jasmine Choi[1], Tanya D'Cruze[6], Eric C. Reynolds[6], Stuart G. Dashper[6], Lynne Turnbull[7], Cynthia B. Whitchurch[7], Timothy P. Stinear[3], Katryn J. Stacey[8] and Richard L. Ferrero[1,2*]

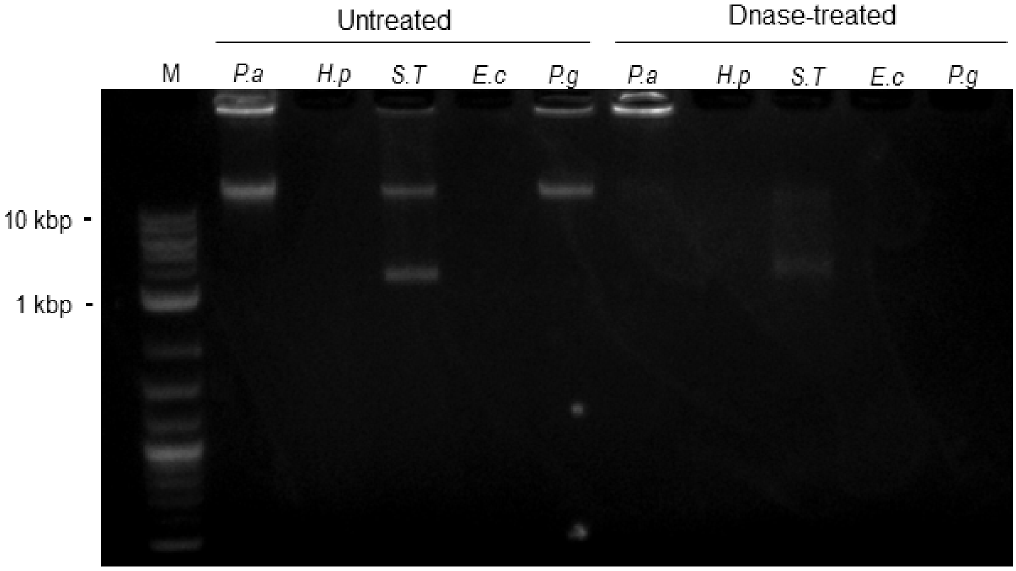

Supplementary Fig. 1 Agarose gel electrophoresis of untreated and DNase treated OMVs from *P. aeruginosa* (*P.a*), *H. pylori* (*H.p*), *S.* Typhimurium (*S.t*), uropathogenic *E coli* (*E.c*) and *P. gingivalis* (*P.g*). M = 2-log ladder marker (NEB).

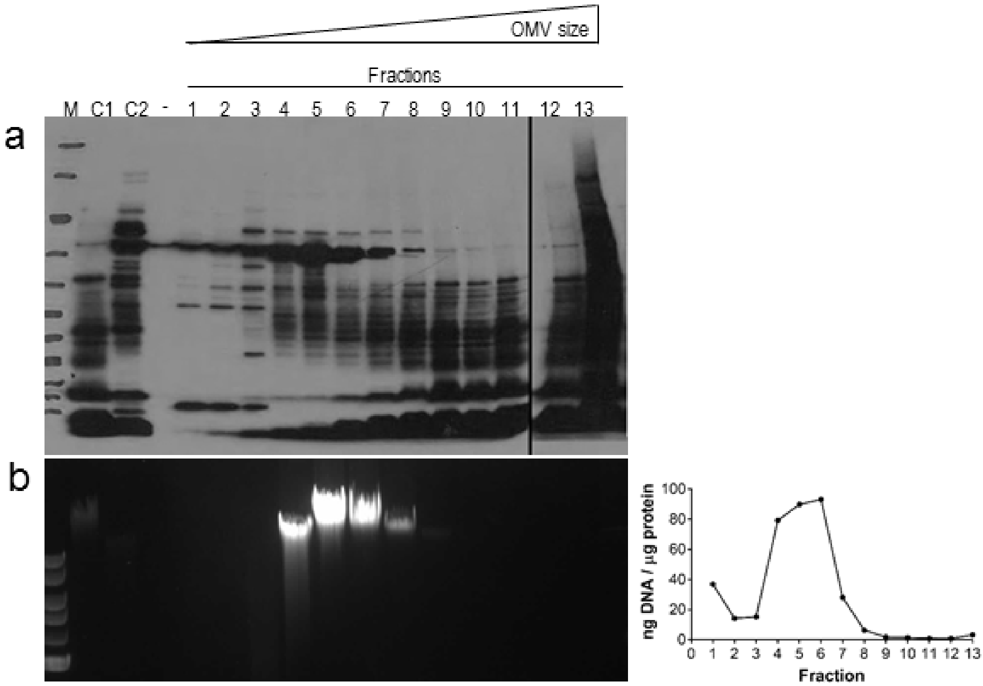

Supplementary Fig. 2 OMVs are associated with high molecular weight DNA. (a) Western blot showing OMVs from *P. aeruginosa* fractionated based on size by sucrose density gradient. Controls include unfractionated OMVs (C1) and *P. aeruginosa* whole cell lysate (C2). Fractions and controls were run on a NuPAGE Bis-Tris 4-12% gel and detection of *P. aeruginosa*-specific proteins was carried out using a polyclonal anti-*Pseudomonas* antibody (Abcam). (b) Agarose gel electrophoresis of *P. aeruginosa* OMV fractions, stained with ethidium bromide, showing high molecular weight DNA associated with OMV fractions 4-9. Quantification of the DNA concentration of each fraction, determined by Qubit dsDNA assay (Life Technologies), is shown.

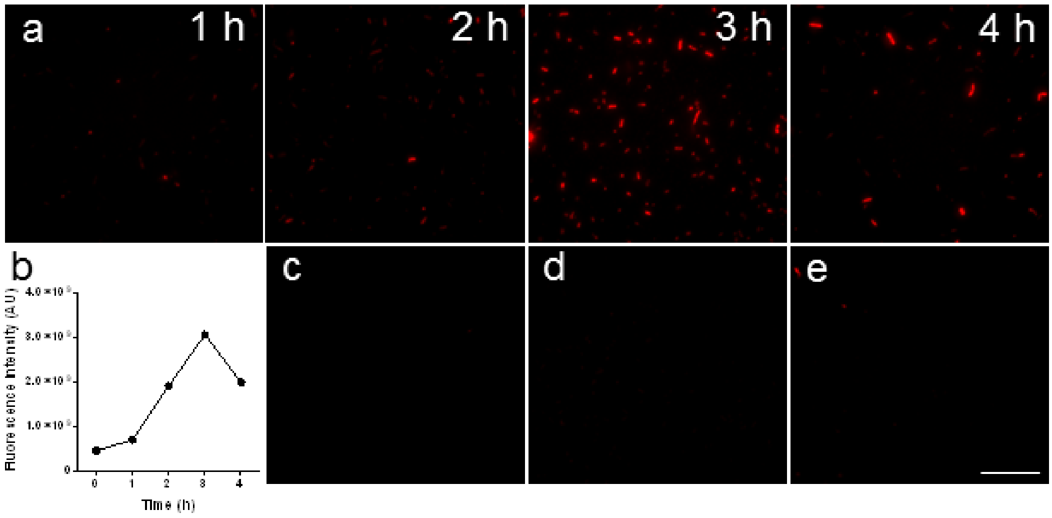

Supplementary Fig. 3 Incorporation of the nucleoside analogue EdU into the gDNA of *P. aeruginosa* bacteria. (a) Cultures were grown in the presence of 1.2 mM EdU for 4 h and aliquots were taken every hour to detect EdU incorporation by fluorescence using biotin azide and AlexaFluor 568-conjugated streptavidin (red). (b) Fluorescence intensity was measured over time, corresponding to EdU incorporation during growth. Controls included (c) cultures grown without EdU, (d) biotin azide omitted and (e) permeabilization omitted. (f) Optimization of biotin azide concentration for detection of EdU incorporation into *P. aeruginosa* DNA with concentrations of biotin azide as shown. Scale bar = 20 µm.

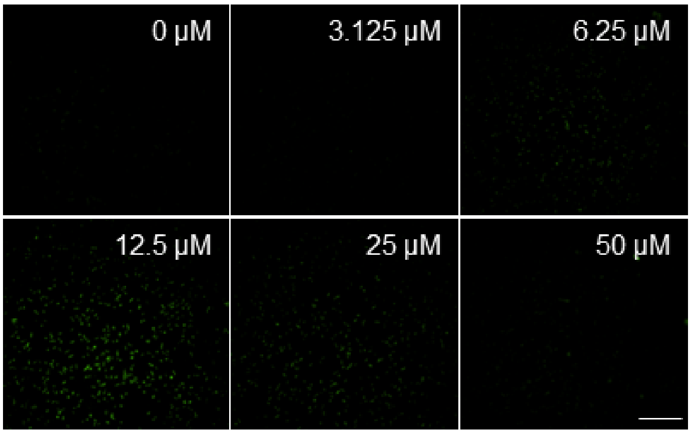

Supplementary Fig. 4 EdU incorporation into *P. aeruginosa* bacteria and their OMVs. (a) Growth curve of *P. aeruginosa* grown in the presence and absence of EdU, showing that EdU does not affect bacterial growth and that the culture was in mid-log phase when harvested at 4 h post inoculation. (b) *P. aeruginosa* OMVs isolated from culture grown in the presence of EdU for 4 h, showing EdU-labelled DNA associated with OMVs. Scale bar = 10 μm.

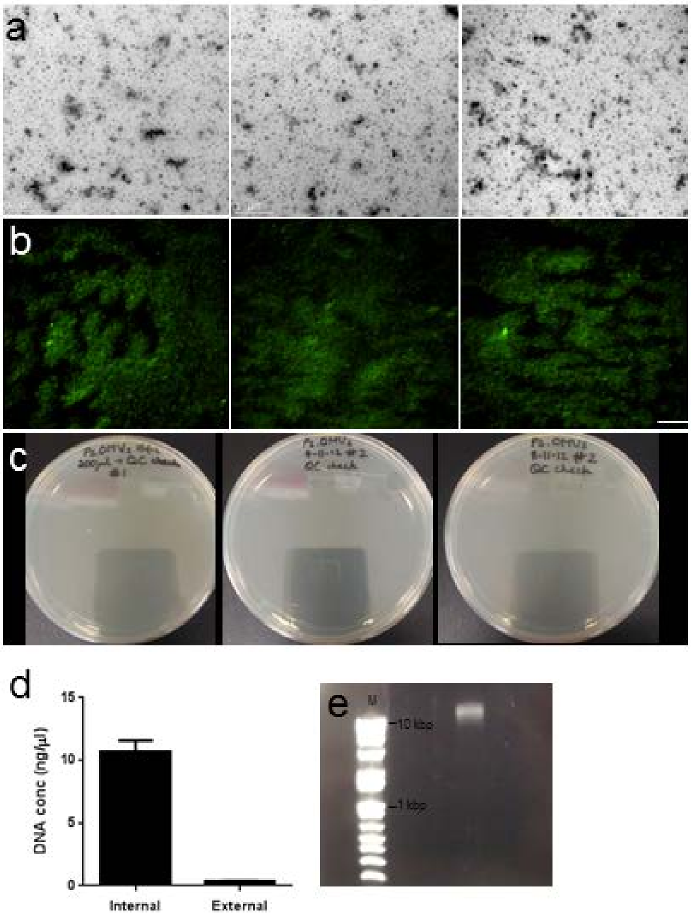

Supplementary Fig. 5 Screening of *P. aeruginosa* OMVs used for sequencing of OMV associated DNA. The absence of viable and nonviable bacteria was determined by (a) EM and (b) confocal microscopy. The three EM and confocal images are representative of 20 fields of view analyzed. Scale bar = 20 µm. (c) The absence of viable bacteria was also verified by the lack of growth on antibiotic free LB agar. (d) Quantification of internal and external DNA in DNase-treated OMVs used for sequencing of internally-derived DNA. (e) Agarose gel electrophoresis of purified, concentrated internally-derived OMV DNA used for sequencing.

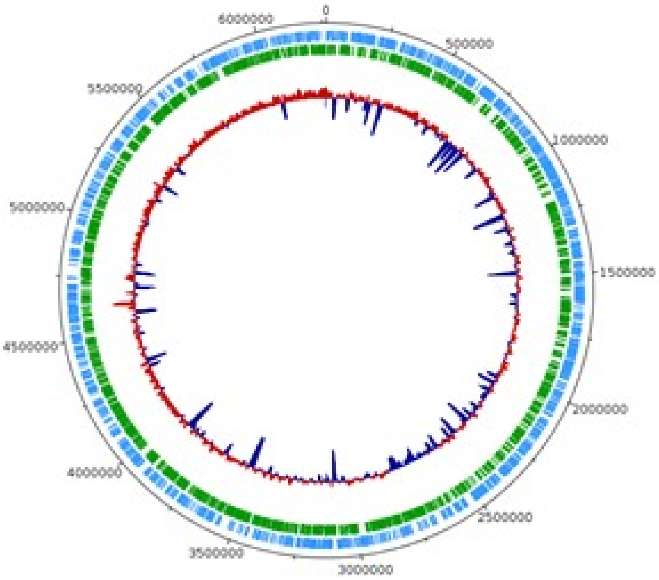

Supplementary Fig. 6 Sequence of the gDNA control from *P. aeruginosa* PA103 (inner circle) plotted against the *P. aeruginosa* PAO1 reference genome (outermost circle; positive and negative sense genes in green and purple, respectively). Positions showing greater than average read density are colored red while those with less than average density are colored blue.

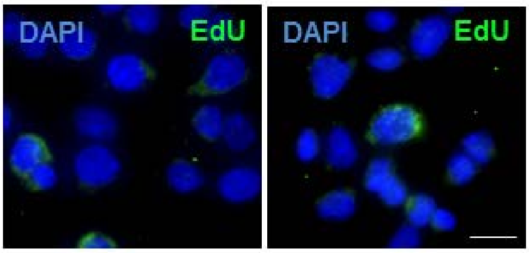

Supplementary Fig. 7 OMV-associated DNA is transported into human gastric epithelial cells (AGS). EdU labelled *P. aeruginosa* OMVs (green) taken up by AGS cells (nuclei blue). Scale bar = 15 µm.

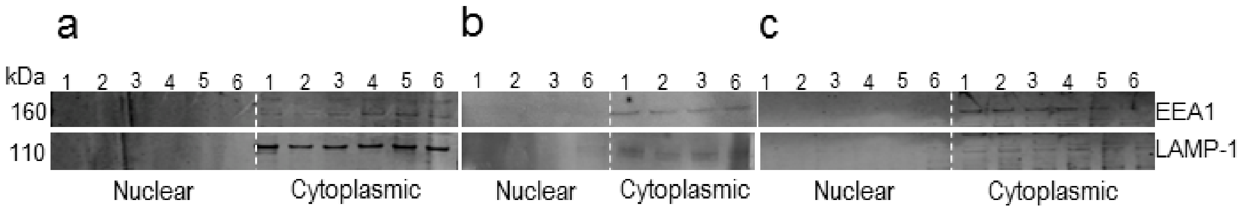

Supplementary Fig. 8 Verification that endosomal compartments are present only in the cytoplasmic extracts. Western blots show early endosomes (EEA1) and late endosomes (LAMP-1) in cytoplasmic extracts of cells untreated (1), treated with intact OMVs (2), DNase-treated OMVs (3), disrupted OMVs (4), free *P. aeruginosa* gDNA (5), or plasmid pGL3c (6), for (a) 4 h, (b) 8 h and (c) 18 h treatments.

Supplementary Table 1. Function and location of genes that were significantly enriched in the internal OMV DNA sequence. A dash is shown where gene symbols are not assigned.

Gene	Function	Location	Ref
prc	Processing of penicillin-binding protein	3642964 - 3645060	(30)
capB	Cold shock protein	3653667 - 3653876	(29)
-	Hypothetical protein	4297250 – 4298227	
-	Probable permease of ABC transporter	4298311 - 4299201	
-	Probable ATP-binding component of ABC transporter	4299204 - 4299998	
-	probable sodium:sulfate symporter	4300117 - 4301949	
-	Conserved hypothetical protein	4302041 - 4303051	
spcS	Chaperone required for secretion of ExoS cytotoxin	4304690 - 4305040	(26)
-	Hypothetical protein	4305645 - 4306256	
-	Probable transcriptional regulator	4306380 - 4307276	
-	Hypothetical protein	4307266 – 4307808	
-	Conserved hypothetical protein	4307912 – 4308382	
-	Hypothetical protein	4308456 – 4309811	
-	Conserved hypothetical protein	4309904 – 4310908	
exoS	Cytotoxin (NAD+ asparagine ADP-ribosyltransferase)	4303141 - 4304502	(25)
-	Hypothetical protein	4310951 – 4311862	
-	Hypothetical protein	4311951 – 4312703	
-	Hypothetical protein	4312707 – 4313642	
-	Probable transferase	4313763 – 4314452	
-	Hypothetical protein	4314481 – 4314795	
-	Hypothetical protein	4314788 – 4315489	
-	Hypothetical protein	4315556 – 4316038	
pcs	Phosphatidylserine synthase (phospholipid synthesis)	4316109 – 4316825	(32)
-	Probable amino acid-binding protein	4316937 – 4317962	
-	Carboxylesterase	4318259 – 4318906	
-	Probable AMP-binding enzyme	4318922 - 4320820	
rhlB	Rhamnolipid biosurfactant synthesis	4321434 – 4322627	(27)
dauB	Predicted ornithine cyclodeaminase	4322788 – 4323735	(31)
dauA	Glycine/D-amino acid oxidases	4323758 – 4324885	(31)
narI	respiratory nitrate reductase gamma subunit	4335276 – 4335959	(28)
narJ	nitrate reductase cofactor assembly chaperone	4335962 - 4336702	(28)
narH	Nitrate reductase beta subunit	4336708 – 4338249	(28)
narG	Nitrate reductase alpha subunit	4338261 – 4342046	(28)
narK2	Nitrate/nitrite transporter	4342122 – 4343528	(28)
narK1	Nitrate/nitrite transporter	4343540 – 4344835	(28)
